# Cryo-EM structure of a natural prion: chronic wasting disease fibrils from deer

**DOI:** 10.1007/s00401-024-02813-y

**Published:** 2024-10-24

**Authors:** Parvez Alam, Forrest Hoyt, Efrosini Artikis, Jakub Soukup, Andrew G. Hughson, Cindi L. Schwartz, Kent Barbian, Michael W. Miller, Brent Race, Byron Caughey

**Affiliations:** 1 Laboratory of Neurological Infections and Immunity, Rocky Mountain Laboratories, Division of Intramural Research, National Institute of Allergy and Infectious Diseases, National Institutes of Health, Hamilton, MT 59840 USA; 2 Research Technologies Branch, Rocky Mountain Laboratories, National Institute of Allergy and Infectious Diseases, National Institutes of Health, Hamilton, MT 59840 USA; 3 Colorado Division of Wildlife, Fort Collins, CO 80526 USA

**Keywords:** Chronic wasting disease, Prion, Deer, Cryo-electron microscopy, Amyloid, Structure

## Abstract

Chronic wasting disease (CWD) is a widely distributed prion disease of cervids with implications for wildlife conservation and also for human and livestock health. The structures of infectious prions that cause CWD and other natural prion diseases of mammalian hosts have been poorly understood. Here we report a 2.8 Å resolution cryogenic electron microscopy-based structure of CWD prion fibrils from the brain of a naturally infected white-tailed deer expressing the most common wild-type PrP sequence. Like recently solved rodent-adapted scrapie prion fibrils, our atomic model of CWD fibrils contains single stacks of PrP molecules forming parallel in-register intermolecular *β*-sheets and intervening loops comprising major N- and C-terminal lobes within the fibril cross-section. However, CWD fibrils from a natural cervid host differ markedly from the rodent structures in many other features, including a ~ 180° twist in the relative orientation of the lobes. This CWD structure suggests mechanisms underlying the apparent CWD transmission barrier to humans and should facilitate more rational approaches to the development of CWD vaccines and therapeutics.

**Supplementary Information:**

The online version contains supplementary material available at 10.1007/s00401-024-02813-y.

## Introduction

Chronic wasting disease (CWD) is an incurable, fatal infectious prion disease that is found in cervid species including deer, elk, moose, and reindeer. It was first identified in a captive deer facility in Colorado in 1967 and has since been detected in more than 30 US states, 5 Canadian provinces, Scandinavian countries, and South Korea [17, 36, 56]. CWD in both farmed and free-ranging cervids has caused substantial economic, ecological, trade, and cultural impacts [12]. With increasing disease prevalence and geographical range, millions of cervids are at risk of being infected. CWD is mainly transmitted by horizontal transmission via infected animals or prion-contaminated environments, but vertical transmission may also contribute [19, 37, 39]. CWD prions released into the environment from both live animals and carcasses can persist as sources of infection for years [34, 38]. Clinical signs include weight loss, behavioral changes, excessive salivation, incoordination, and weakness. The natural disease course is measured in years, although animals may survive for only weeks to months after the onset of clinical signs.

Prion diseases are lethal with no approved vaccines or treatments, making them problematic in natural, agricultural, and clinical settings. The transmission and fatal consequences of bovine spongiform encephalopathy (BSE) prions to humans via consumption of infected meat [8] have heightened concerns that CWD might be similarly transmissible to humans. However, so far, no CWD prion transmissions to humans have been established, and no causal link between CWD prevalence and incidence of prion disease has been observed based on surveillance data from CWD endemic areas. In vitro tests and experimental transmissions of CWD from cervids into animal models such as non-human primates or mice expressing human PrP^C^ have usually indicated a substantial, but not necessarily impenetrable, CWD transmission barrier between cervids and humans (reviewed in [42, 46]). Humans may be protected from CWD by factors such as molecular incompatibility between cervid PrP^Sc^ and human PrP^C^ [42, 46], the natural routes and doses of exposure, and immune responses to heterologous inocula. Nonetheless, CWD strain variation or passages through intermediate non-cervid species—should they occur—might abrogate these protections. Improved understanding of CWD prion structural details may provide insights into the mechanisms underlying CWD transmission barriers and the relative risks of CWD transmission to non-cervid species.

We and others have recently solved cryo-EM-based structures of multiple strains of fully infectious brain-derived PrP^Sc^ prions from hamsters and mice [20, 21, 25, 31, 32]. These experimental prion strains were adapted to rodents from sheep scrapie. Each of these rodent-adapted strains contains single-protofilament amyloid fibrils that have broadly similar parallel in-register intermolecular β-sheet (PIRIBS)-based ordered cores spanning from residues 93–95 to 225–230. The cores of the rodent prion fibril cross-sections contain amino-proximal (N), middle, and disulfide arches as well as a steric zipper that holds the extreme N-terminal residues of the core against the head of the middle arch. These features form major N- and C-terminal lobes of the fibril cross-sections. However, detailed conformations and relative orientations of these common structural elements differ markedly between strains, providing distinct templates for prion growth and a conformational basis for the propagation of distinct strains. Of note, these rodent species are not known natural hosts of prion diseases. Amyloid fibrils isolated from humans with the F198S *PRNP* genotype of Gerstmann–Straussler–Scheincker syndrome (GSS) have a 2–4 protofilament PIRIBS architecture with the ordered core of each protofilament comprising a much shorter span of residues (80–141) [16] than the rodent prions.

Here we report a 2.8 Å cryo-EM structure of CWD prion fibrils from brain tissue of a naturally infected white-tailed deer (*Odocoileus virginianus*). This structure, like previously reported prion structures, contains a PIRIBS-based core but other structural details differ strikingly from the known rodent and mutant human prion structures. Knowledge of this natural CWD prion structure provides a molecular basis for the pursuit of vaccines, therapeutics, and the understanding of molecular mechanisms of transmission barriers.

## Materials and methods

### Tissue and CWD fibril purification

Brain tissue was obtained from a symptomatic white-tailed deer provided by Montana State Department of Fish, Wildlife and Parks in compliance with their jurisdiction. CWD fibrils were purified from brainstem tissue using a previously described protocol [25] except for the following minor modifications: A 20% w/v homogenate was made with phosphate buffered saline using 10 g of the cortex region. Volumes of 25% sarkosyl and 10X TEND buffer (which, at 1X is 10 mM Tris-HCI, pH 8.0 at 4 °C, 1 mM EDTA, 133 mM NaCl, 1 mM dithiothreitol) were added to give final concentrations of 2% sarkosyl and 1X TEND. A Roche cOmplete, EDTA-free protease inhibitor tablet was also added to the brain lysate before performing the remaining steps as described [25]. The final fibril preparations (0.21 mg/ml protein) were vortexed and allowed to sit for several minutes to pellet highly bundled fibrils. Aliquots from the supernatant fraction were diluted in 20 mM Tris pH 7.4, 100 mM NaCl, vortexed, and then supplemented with 0.02% amphipol 8–35 and sonicated immediately prior to grid preparation.

### RT-QuIC assay of CWD prion

End-point dilutions of the purified CWD fibril preparation were performed using the RT-QuIC assay with recombinant hamster PrP 90–231 substrate to measure prion seeding activity. The RT-QuIC master mix composition was 10 mM phosphate buffer (pH 7.4), 300 mM NaCl, 0.1 mg/mL PrP, 10 µM thioflavin T (ThT), and 10 mM EDTA. Reaction buffer (98 µl per well) was loaded into a black 96-well plate with a clear bottom (Nunc). 2 µl of purified CWD fibrils (0.84 mg/ml), normal brain homogenates, or serial tenfold dilutions thereof in 0.1%SDS in phosphate buffered saline with 130 mM NaCl and 1X N2 supplement (Gibco), were used to seed quadruplicate reactions. The plates were sealed with a sealing tape (Nalgene Nunc) and then incubated in a BMG Fluostar plate reader at 50 °C for 30 h with cycles of 1 min shake (700 rpm double orbital) and 1 min rest throughout the incubation. ThT fluorescence measurements were done at 450 ± 10 nm excitation and 480 ± 10 nm emission.

### Negative stain electron microscopy

3 µl of sample was added to glow-discharged ultrathin carbon on lacey carbon support film grids (400 mesh, Ted Pella, Redding, CA) for 1 min, briefly washed with dH20, then negatively stained with Nano-W (2% methylamine tungstate) stain (Nanoprobes, Yaphank, NY). Grids were imaged at 80 kV with a Hitachi HT-7800 transmission electron microscope and an XR-81 camera (Advanced Microscopy Techniques, Woburn, MA).

### Cryo-EM grid preparation

Quantifoil 1.2/1.3 200 mesh copper grids (Electron Microscopy Sciences, Hatfield, PA) were glow-discharged with an oxygen/hydrogen mixture in a Solarus 950 (Gatan, Pleasanton, CA) for 10 s. After mounting a grid in an EM GP2 plunge freezer (Leica, Buffalo Grove, IL), a 3 μl droplet of 0.02% amphipol A8-35 in phosphate buffered saline was added to the carbon surface and hand blotted to leave a thin film. The grid was then raised into the plunge freezer chamber, which was set to 22 °C and 90% humidity. 3 μl of sample that had been recently sonicated in a cup horn was delivered to the carbon side of the grid and allowed to sit for 60 s. Liquid was then blotted from the sample for ∼ 4 s followed by a 3 s drain time before plunge freezing the grid in liquid ethane at − 184 °C. Grids were then mounted in AutoGrid assemblies.

### Cryo-EM tomography

Grids were prepared as for single-particle imaging, except that before adding sample to the grid, the sample solution was diluted with 1/3 volume of a 5 nm gold fiducials solution as provided by the manufacturer (Cat. # FG 5 nm; CMC, Utrecht, The Netherlands). Tilt-series were acquired using Tomo5 on a Titan Krios G4 (Thermo Fisher Scientific, Waltham, MA) operating at 300 keV on a K3 direct electron detector with a Biocontinuum energy filter (Gatan, Pleasanton, CA) with a slit width of 20 eV. Tilt-series were acquired at 81,000 X (0.5289 Å pixel), ± 60°, every 3° in a dose-symmetric fashion [15]. Total dose was ~ 90 e/Å^2^ with ~ 2.3 e/Å^2^ per tilt. Defocus ranged from -8 to -10 µm. Tomograms were reconstructed using weighted back projection with IMOD [26], followed by a nonlinear anisotropic diffusion filter [14] for visualization of fibril twist.

### Single-particle acquisition

Grids were loaded into a Krios G1 (Thermo Fisher Scientific, Waltham, MA) transmission electron microscope operating at 300 kV equipped with a K3 detector (Gatan, Pleasanton, CA) and a Biocontinuum GIF (Gatan, Pleasanton, CA) operating at a slit width of 20 eV. 79,996 non-gain normalized tif movies were acquired at a pixel size of 0.828 Å with SerialEM [33] at a nominal magnification of 105,000 X. Total dose per movie was ~ 56 e/Å^2^ with ~ 1 e/Å^2^/frame and with a dose rate of ~ 15 e/pix/sec. Defocus values were set to cycle multiple steps between − 0.5 and 2 µm.

### Helical reconstruction

Motion correction of raw movie frames was performed with RELION 5.0 beta [51]. CTF estimation was performed using CTFIND4.1 [48]. All subsequent processing was performed in RELION. Fibril start-to-end coordinates were picked manually, and segments were extracted with an inter-box distance of 23.75 Å using a box size of 1024-pixels. Segments from the large box size were used to estimate the fibril cross-over distance and for estimating initial twist parameters. 2D classes from the long segments were used to generate an initial 3D model.

The particles were re-extracted with 384-pixel boxes. After 2D classification, suboptimal classes were removed. 3D auto-refinement was performed on particles from suitable 2D classes. Following 3D auto-refinement, 3D classification was performed without allowing for image alignment to further remove suboptimal particles. Classes displaying well aligned segments, from auto-refinement, were selected for further refinement. Refinement of the helical twist and rise resulted in a twist of − 0.291° and rise of 4.779 Å. Iterative cycles of CTF refinement and Bayesian polishing were used until resolution estimates stabilized. Post-processing in RELION was performed with a soft-edged mask representing 10% of the central Z length of the fibril [29] yielding a 2.8 Å map. Resolution estimates were obtained between independent refined half-maps at 0.143 FSC. The map was density modified using Resolve Cryo EM [52] and then helical symmetry was applied in real-space to improve visualization for model building and refinement.

### Model refinement

The CWD atomic model, comprising the protease-resistant core residues 92 through 229, were built de novo using Coot [13]. The initial beta-rung was expanded to 6 subunits for subsequent refinement. Real-space refinement using Phenix [1, 18] and Fourier space refinement using RefMac5 were performed iteratively with subsequent validation. Secondary structure and NCS restraints were included during iterative refinement. Model validation was performed with CaBLAM [55], MolProbity [10], and EMringer [3], and any identified outliers/clashes were corrected for subsequent iterative refinements/validation.

## Results

### CWD PrP^Sc^ purification and prion seeding activity

**Fig. 1:**
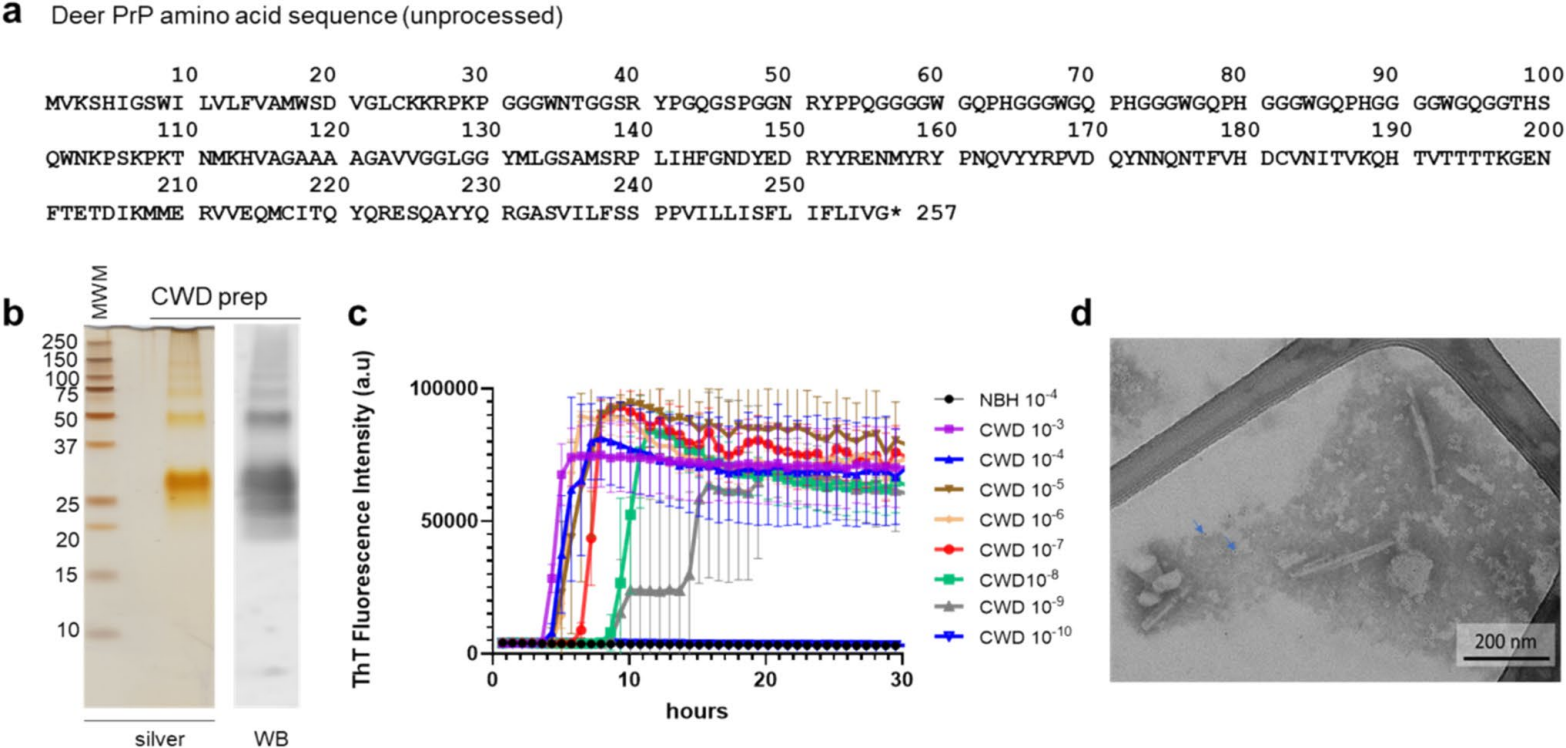
PrP amino acid sequence, purity, seeding activity, and morphology of CWD PrP^Sc^ preparation. **a** PrP amino acid sequence determined for the white-tailed deer source of our CWD brain tissue before cellular processing to remove N-terminal signal peptide and C-terminal GPI anchor signal. **b** Silver-stained SDS-PAGE gel and western blot of CWD PrP^Sc^. **c** End-point dilution RT-QuIC analysis of purified CWD prion preparation. Traces show mean ± SD of ThT fluorescence from quadruplicate RT-QuIC reactions seeded with serial tenfold dilutions of the preparation (as indicated) over time. Negative control reactions were seeded with normal brain homogenate (NBH) at 10^–4^ w/v tissue dilution. **d** Negative stain transmission EM of the CWD PrP^Sc^ preparation showing amyloid fibrils. Smaller circular structures with dense centers were also seen (arrows mark examples), which were presumably ferritin, a previously observed contaminant of PrP^Sc^ preparations [4] that would not be confused with amyloid fibrils in the cryo-EM analyses

We isolated proteinase K (PK)-resistant PrP^Sc^ from the brain of a CWD-infected white-tailed deer (WTD; *Odocoileus virginianus*) from Montana that expressed the most common WTD PrP variant (Q95, G96, S100, N103, A123, Q226, Q230; Fig. 1a) as determined by Sanger sequencing of its *PRNP* gene [50]. Analysis of the proteinase K-treated CWD PrP^Sc^ preparation by SDS-PAGE with silver staining or western blotting indicated that its protein content was largely PrP and that the banding profile was consistent with prior reports [25] with differentially glycosylated bands at ~ 20–32 kDa and SDS-resistant oligomers thereof (Fig. 1b). The infectivity of CWD fibrils was estimated using the RT-QuIC prion seed amplification assay. The endpoint dilution, at which less than all quadruplicate wells were ThT positive, was ~ 10^–9^ (Fig. 1c) giving a prion seed concentration (i.e., the concentration of seeding units giving 50% positive replicate RT-QuIC reactions, or SD_50_) of ~ 12 logSD_50_/mg of purified protease-resistant PrP^Sc^ preparation. This seeding activity was similar to our prior ex vivo isolates of prions from rodent hosts, which were also shown by in vivo bioassay to be highly infectious [20, 21, 25]. Negative stain EM indicated a primarily fibrillar morphology with a mixture of individual, laterally associated, and crossed fibrils (more isolated fibrils shown in Fig. 1d).

### Cryo-EM, single-particle analysis, and helical reconstruction of 3D density map

**Fig. 2:**
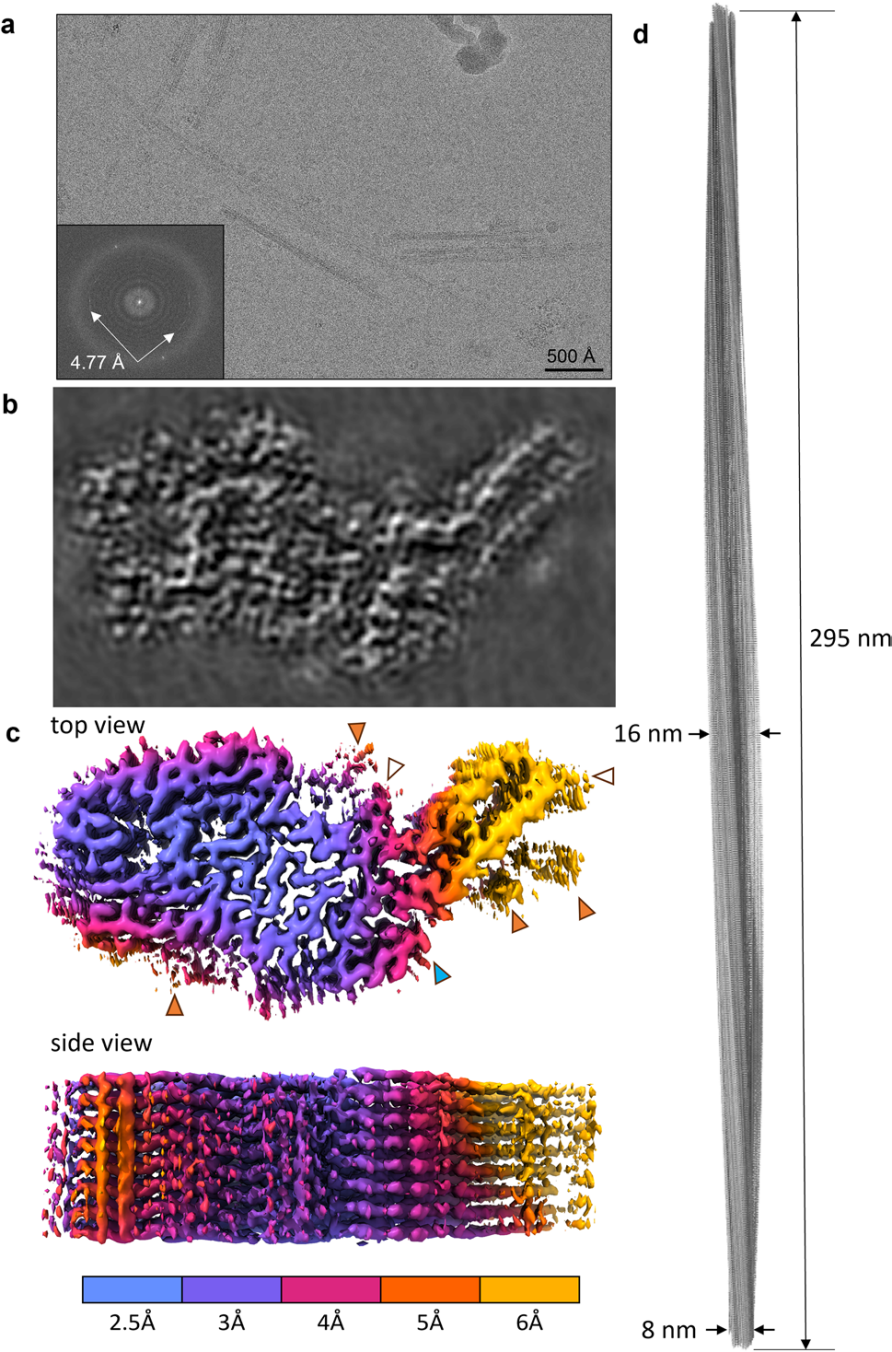
Cryo-EM images and density maps of CWD prion fibrils.** a** Representative 2D cryo-EM image of unstained CWD fibrils in vitreous ice layer. Inset: fast Fourier transform of 2D image showing signals from regular 4.77 Å spacings (arrows). **b** Projection of density map of fibril cross-section derived from single-particle cryo-EM analysis. **c** Surface depictions of density map with colors showing local resolutions according to color bar. Orange arrowheads: peripheral densities not attributed to polypeptide in the subsequent modeling; open arrowheads: the sites of potential N-linked glycans; blue arrowhead: C-terminus where GPI anchor is attached. **d** Elongated projection of the fibril density map representing a 180° twist along the axis (i.e., the cross-over distance of 295 nm)

Unstained CWD prion fibrils were embedded in vitreous ice and imaged using cryo-EM single-particle acquisition and helical reconstruction approaches. The associated parameters are given in Methods and Table 1. We observed fibrils that were either individual, crossing over one another, or laterally bundled (Fig. 2a). Other less-distinct clumps of unidentified material were also sometimes seen. Start–end coordinates were manually picked from discreet fibrils that were not bundled and initial particles were extracted at 23.75 Å intervals. Fast Fourier transforms of the image in Fig. 2a indicated regular axial spacings of ~ 4.77 Å (inset) as was observed in rodent-adapted prions, consistent with the spacing of rungs of β-sheets. 2D class averages were obtained from a total of 535,858 particles (Table 1). The 2D class averages showed cross-over points and twisting axial bands of density along the fibril axis. The degree of twist was much less than that seen with previous brain-derived prion fibrils [20, 21, 25, 31, 32], but cryo-EM tomography indicated left-handed twist in all 24 fibrils analyzed from 14 tomograms (Fig. S1 and Table S1). Striations perpendicular to the fibril axis became visible in images of 2D class averages, with Fourier transforms again indicating ~ 4.77 Å axial spacing (Fig. S2b). 3D classification converged on a single core morphology (Fig. 2b, d and S3). Using helical reconstruction, we obtained an overall resolution of 2.8 Å for the CWD fibril core (Fig. S2c). As has been typical of rodent-adapted prion fibrils [20, 21, 25, 31, 32], lower resolution was seen at the tip of the structure that corresponds to the disulfide arch in the atomic model (see below). Again, rungs running perpendicular to the fibril core were stacked along the fibril axis at a spacing of ~ 4.77 Å (Fig S2b and 2d).

**Table 1:** Cryo-EM data, refinement, and validations

Data collection and processing	CWD	CWD with NAG*
Magnification	105,000x	105,000x
Voltage (kV)	300	300
Electron dose (e-/Å^2^)	55	55
Calibrated pixel size (Å)	0.8283	0.8283
Symmetry imposed	C1	C1
Initial particle segments	535,858	535,858
Final particle segments	7346	7346
Map resolution (Å)	2.8	2.8
Helical rise (Å)	4.779	4.779
Helical twist (°)	− 0.2913	− 0.2913
Map sharpening B factor	− 22	− 22
Model refinement		
R.M.S deviations		
Bond lengths (Å)	0.002	0.002
Bond angles (°)	0.481	0.459
MolProbity score	1.52	1.54
Clash score	9.91	10.79
Rotamer outliers (%)	0.0	0.0
Ramachandran plot		
Favored (%)	98.53	98.53
Allowed (%)	1.47	1.47
Outliers (%)	0	0
EM Ringer score	3.66	3.82
Model vs. Data (CC)	0.78	0.77

*Additional information regarding model that includes the first N-linked glycan units at N184 and N200 (NAG: N-acetylglucosamine)

### Atomic model of the CWD prion

We used the final reconstructed 3D density map to build an atomic model of the CWD fibril using the PrP polypeptide sequence of residues 92–229, a span of residues consistent with the known protease-resistant core of the CWD prion [58]. Table 1 provides parameters of our iterative real and Fourier space refinements and validation.

**Fig. 3:**
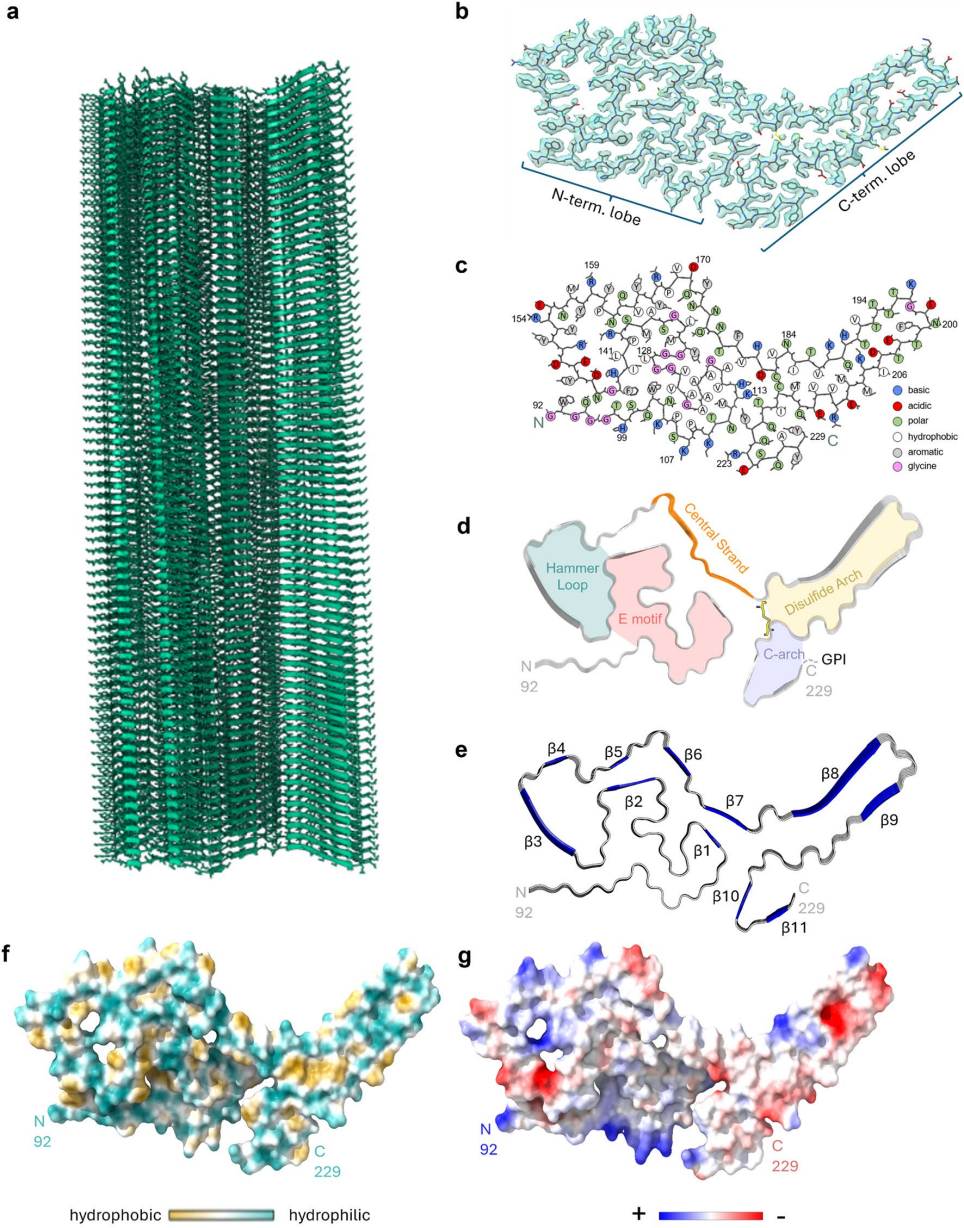
CWD prion model based on cryo-EM density map.** a** Extended fibril model as a ribbon diagram. **b** PrP residues 92–229 threaded through a cross-sectional density map. **c** Schematic depiction of fibril core residues showing sidechain orientations relative to the polypeptide backbone. **d** Ribbon cartoon of the fibril cross-section to identify structural elements as labeled. **e** Stacked hexameric segment of the fibril with β sheets. **f** Relative surface hydrophobicity as indicated in the color bar. **g** Coulombic charge representation. The analyses in **f** and **g** were performed using ChimeraX 1.4

**Fig. 4:**
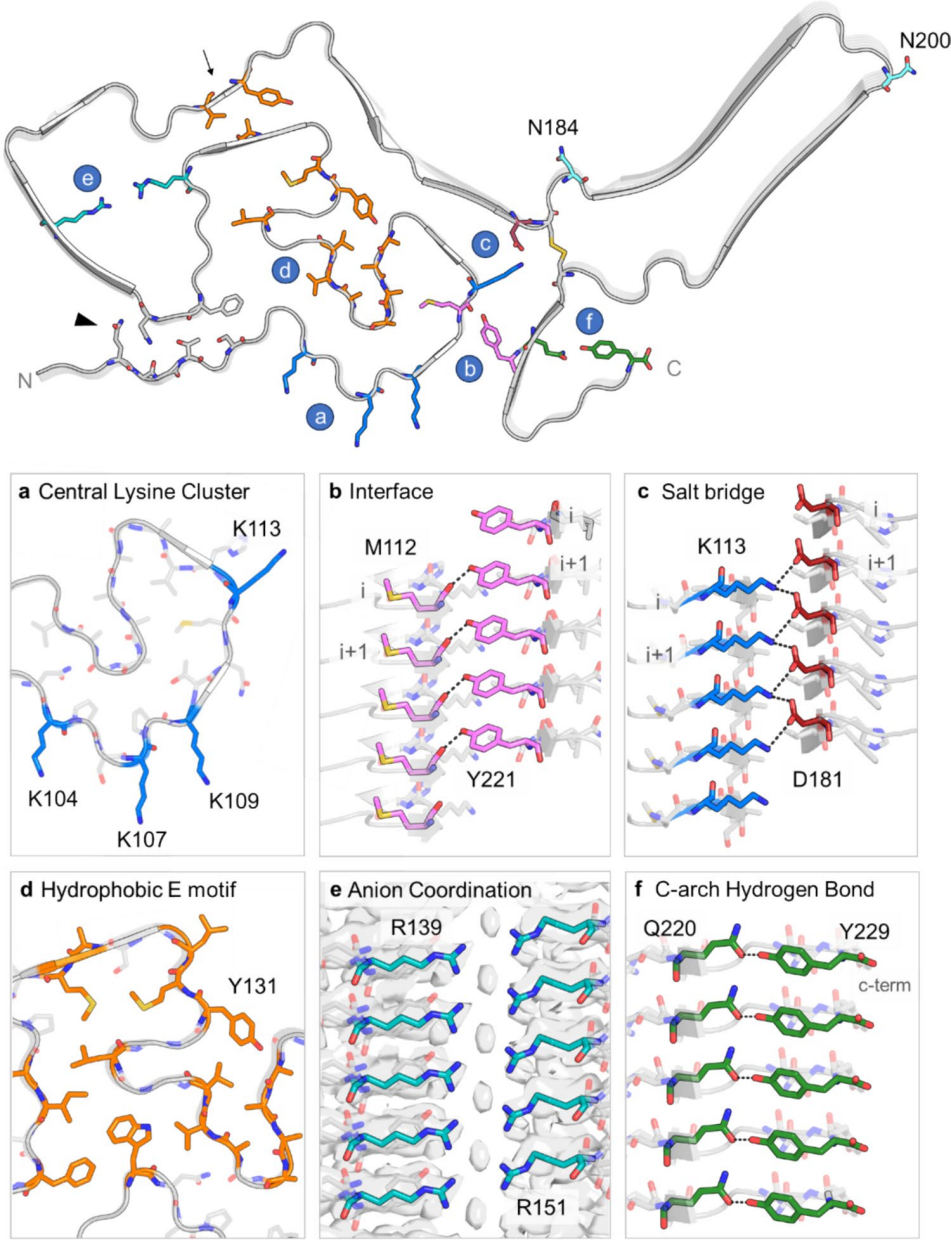
Core elements of CWD PrP^Sc^ displayed on the backbone of the fibril (top). The letters in blue circles correspond to the individual panels. Residues N184 and N200 mark the N-linked glycosylation sites. An arrow points to hydrophobic residues in the neck of the hammer loop. An arrowhead marks N95, the stacked sidechains of which along the fibril axis form an amide zipper. **a** Cross-sectional view of central lysine cluster encompassing residues K104, K107, K109, and K113 (blue). **b** Side view of hydrogen bond in the interface of the N- and C-lobes formed by M112 and Y221. **c** Side view of stabilizing salt bridge formed between K113 and D181. **d** Cross-sectional view of hydrophobic residues (orange) in the E motif. **e** Side view of ambiguous cryo-EM density between positively charged residues R139 and R151. **f** Side view of hydrogen bond between Q220 and Y229 at the top of the C-arch

As with the structures of the known rodent-adapted prions [20, 21, 25, 31, 32], the CWD fibril cross-section comprised a single PrP molecule with major N- and C-terminal lobes (called N- and C-lobes, respectively). We have defined major motifs comprising the N-lobe as the E motif (in reference to its shape), hammer loop (also shape), and central strand, while the C-lobe contains the disulfide arch and C-arch (Fig. 3d). As in the rodent strains, there was preferential exposure of cationic sidechains in the N-terminal half and anionic sidechains in the C-terminal half of the cross-section (Fig. 3g). Large contributors of the cationic regions in the N-lobe were the central lysine cluster (CLC; residues K104, K107, K109 and K113), and positively charged residues in the hammer loop (Fig. 3c, d). Interestingly, the CLC of the CWD fibril was not completely as solvent-exposed as the CLC in the rodent PrP^Sc^ structures (Fig. 4a). Moreover, we observed densities adjacent to the axial stacks of cationic side chains along the N-terminal half of the core (Fig. 2c, orange arrowhead), all of which were presumably too variable in structure to be resolved. It is likely that these ambiguous densities reflect either remnants of the more extreme N-terminal PrP sequence after partial proteolysis or non-PrP ligands.

Peripheral densities were also observed adjacent to the N-linked glycosylation sites N184 and N200 (Fig. 2c, open arrowheads; and Figs. S3, S4). These densities were consistent with the first N-acetylglucosamine residue of the glycan chains and were partially resolved (> 6 Å resolution) as shown in Fig S4. Interestingly, the first glycosylation site is nestled in the cleft between the N- and C-lobes which has a width of ~ 60 Å, enabling the glycan to potentially interact with solvent-exposed residues 174–183. More distal components of the glycan chains on other strains of PrP^Sc^ have been found to be highly variable in structure [49] and, thus, poorly resolved in previous cryo-EM prion structures [20, 21, 25, 31, 32].

**Fig. 5:**
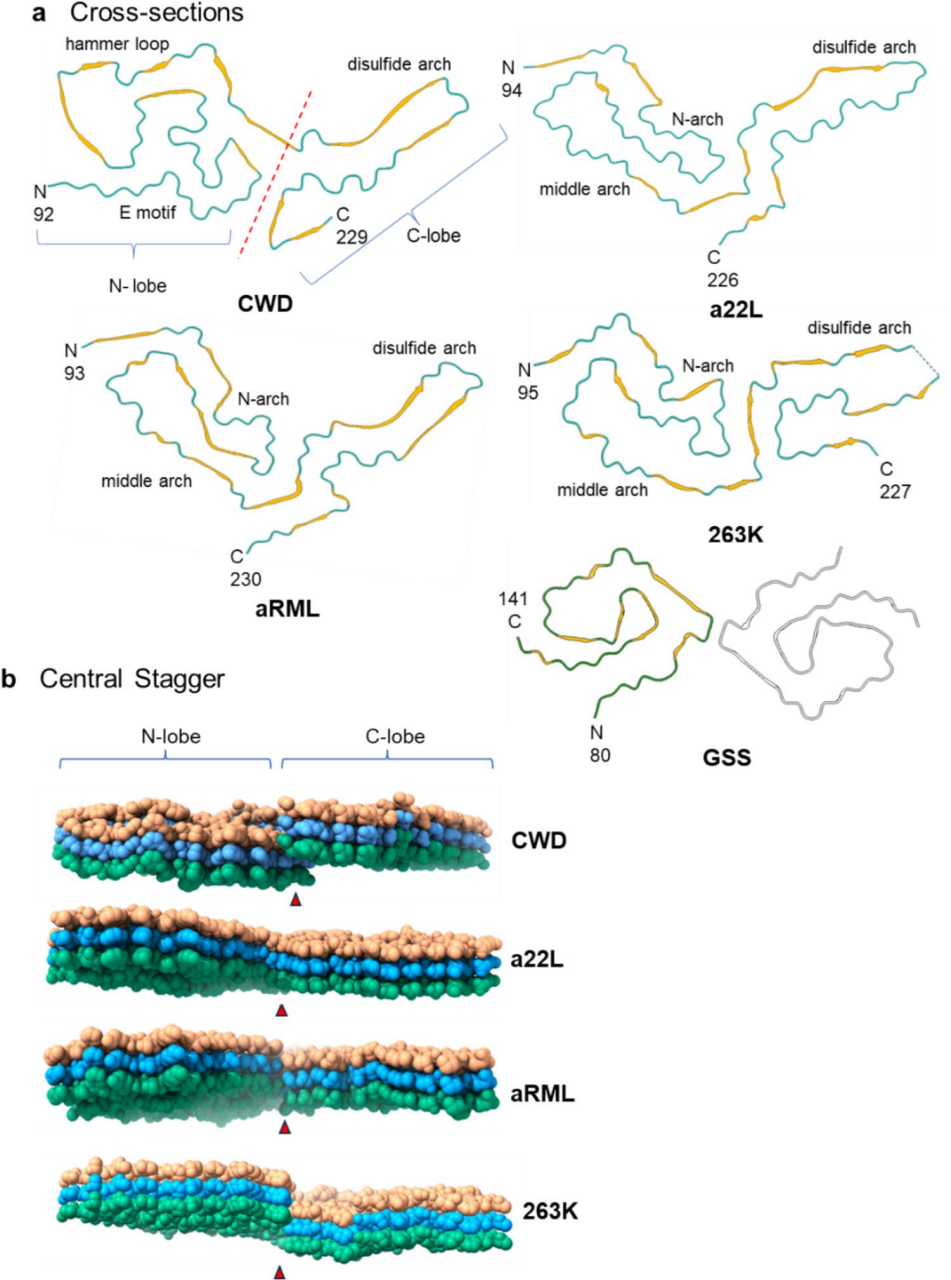
Comparison of CWD to rodent-adapted prion strains. **a** Ribbon diagrams of monomers within CWD (this paper), a22L [20], aRML [21], 263K [25], and GSS F198S [16] fibril core structures. The 2-protofilament subtype of the GSS fibril is depicted, with the second protofilament shown in grey. The red dashed line separates the N- and C-lobes of the CWD cross-section. **b** Lateral views of space-filling models of stacks of three monomers of CWD, a22L, aRML, and 263K EM models. The orientations of these views relative to the ribbon diagrams in **a** are as if looking at the latter from below. Thus, whereas the C-lobes of each model in **b** show the side with the exposed C-terminus, the orientation of the CWD N-lobe is swiveled 180° relative to that of the other N-lobes. Arrowheads indicate interface between the head of the N- and C-terminal lobes of a given monomer

**Fig. 6:**
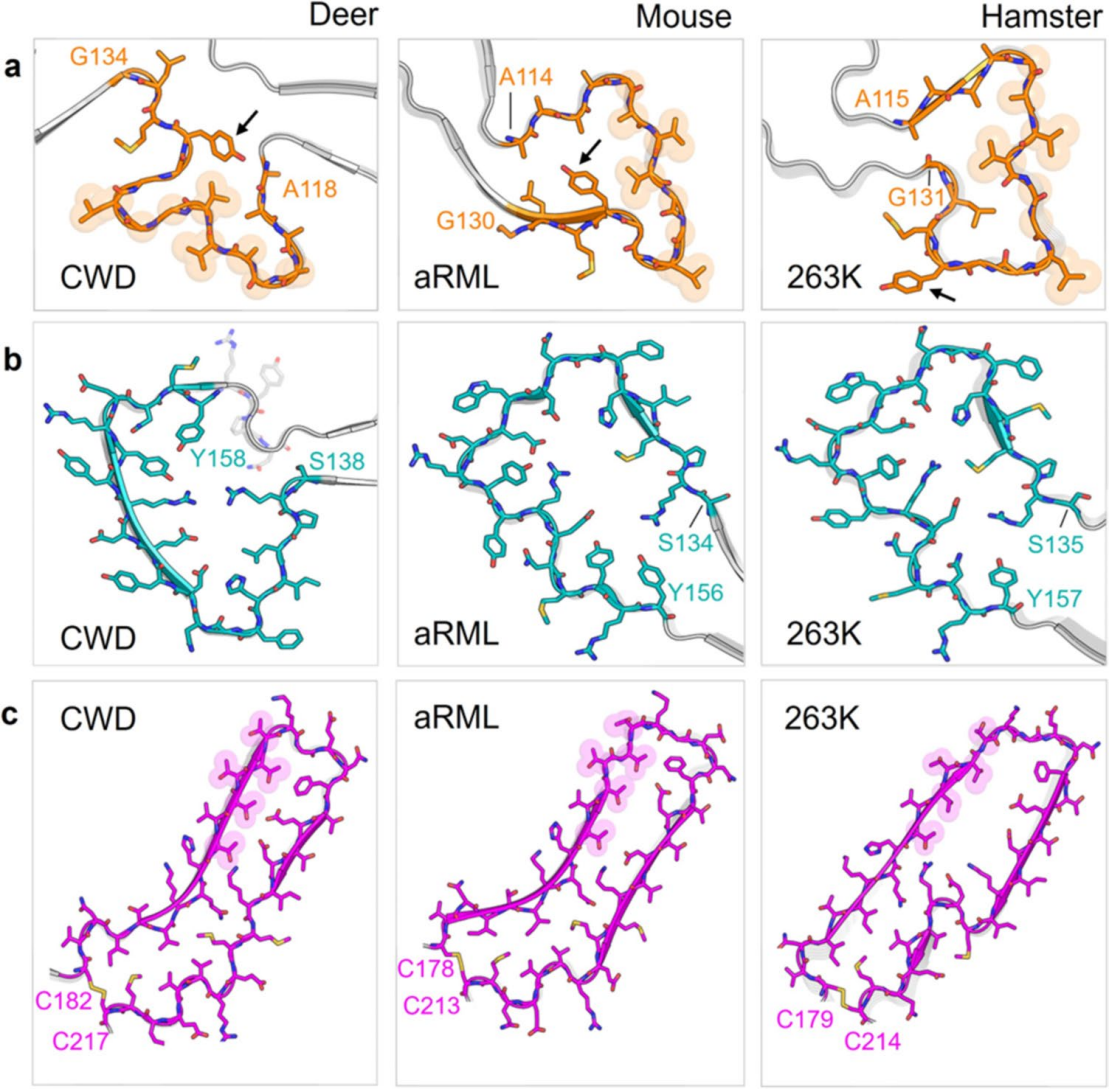
Comparison of major structural motifs between deer CWD and rodent-adapted scrapie strains.** a** Residues ~114–134, which are part of the E motif in CWD and comprise the N-arch in both mouse (aRML) and hamster (263K) strains. Arrows mark tyrosine 131 (Y127 in aRML; Y128 in 263K). **b** Residues 135–157, which are part of the hammer loop in CWD and the middle arch in both mouse and hamster strains. **c** The disulfide arch, which begins at the corresponding disulfide bridges of the CWD, aRML, and 263K fibrils

The N-lobe of the CWD fibril was quite distinct from those of the rodent-adapted prions, both in the complex convolutions of the polypeptide backbone and in the ~ 180° rotation relative to the C-lobe within the plane of each rung. In the N-lobe, the E motif was largely stabilized by hydrophobic amino acids and displayed some similar structural elements (between residues 118 and 134) to those observed in the N-arch of the rodent-adapted PrP^Sc^ strains (Fig. 4d, Fig. 6a, see Discussion). The hammer loop was wide enough to create a pore along the axis of the fibril, with a 5.7 Å gap between the opposing sidechains of R139 and R151 in the model. Interestingly, the cryo-EM map had an ambiguous density between these positively charged sidechains (Fig. 4e). Although the identity of the density was not clear, it is likely to be a small anion. Hydrophobic interactions between the top of the E motif and the end of the hammer loop constricted the region preceding the central strand (Fig. 4, arrow). The β-strands (β1 and β7) in the more C-terminal portion of the central strand were terminated by a salt bridge formed between residues D181 and K113 (the only buried lysine of the CLC) (Fig. 4c). The individual rungs of the CWD fibril were not entirely coplanar, which created a staggered interface between the N- and C-lobes formed by residues 109–113 in the N-lobe, and 219–223 in the C-lobe (Figs. 4b, 5b). The cleft between the N- and C-lobes was gated by two positively charged residues on either side (K109 and R223), and the interface between the two lobes was stabilized by a hydrogen bond formed between the backbone carboxyl of M112 (internally facing) and the phenolic hydroxyl group of Y221 (Fig. 4b).

One motif that is present in all the PrP^Sc^ fibril structures to date is the disulfide arch. At its base, the CWD fibril contained a disulfide bond between C182 and C217 (Fig. 3b, d), which was surrounded by a patch of hydrophobic residues (Fig. 3f). The overall shape and width of the CWD disulfide arch were most similar to that of the murine wild-type and anchorless (a) RML strains (Fig. 6c, Fig. S5d). Interestingly, the top of the disulfide arch contained almost five consecutive threonines (191,193–196), with three internal and two solvent-exposed. This stretch of threonines likely contributes to the flexibility in the backbone, as seen by its less-resolved density in the cryo-EM map (Fig. 2c). Most of the charged residues in the disulfide arch were solvent-exposed, including lysine (K197) and glutamic acid (D199) on the tip of the arch. The three buried charged residues were E203, D205, and K207. To compensate for charge, the aspartic acid (D205) likely forms a salt bridge with neighboring K207. However, the positioning of E203 precluded any proximal charge-neutralizing interactions. This suggests a possible hydration pocket or that the glutamic acids have an anomalous pK_a_. The C-arch (residues 215 to the C-terminus at 229) was stabilized by a hydrogen bond which spanned across the width of the arch between residues Q220 and Y229 (Fig. 4f).

## Discussion

### Comparisons to other types of pathogenic amyloids

CWD appears to be more naturally and casually infectious than most other prion or prion-like proteinopathies. This infectivity makes the CWD structure an important point of reference in understanding features governing relative transmissibilities within the broad spectrum of pathogenic protein aggregates of humans and other mammals [9, 24]. Much remains to be established about the features that make CWD so infectious. However, two striking features that distinguish CWD and most other highly infectious PrP prions from other types of pathological amyloids are the arrays of N-linked glycans and GPI anchors projecting outward from their fibril cores. The glycans may help to protect the assemblies from proteostatic clearance mechanisms. The GPI anchors presumably mediate membrane interactions, and such interactions may facilitate the binding of inoculated prions to cells, as well as the uptake, fragmentation, and transport of prions within and between cells via membranous structures such as endosomes, transport vesicles, exosomes, and tunneling nanotubes. Most natural infections are likely to occur via peripheral sites of exposure, and efficient spreading of prion infections from such sites to the brain can require GPI-anchored PrP^C^ in neurons [23]. Although prions can spread within the brain in transgenic mice expressing only GPI-anchorless PrP after localized intracerebral inoculation [44], the incubation periods required to reach the terminal stage are usually much longer than in wild-type mice [11]. Sigurdson and colleagues have reported that incubation periods can be markedly reduced with serial intracerebral passages of prions in anchorless PrP transgenic mice [7], but this is likely due in large part to the need to accumulate much higher loads of anchorless PrP^Sc^ in the brain to cause terminal disease. Thus, serial passages of a set amount of terminal brain tissue would involve the transfer of commensurately higher amounts (i.e., titers) of PrP^Sc^, thus reducing incubation periods even if anchorless, and underglycosylated, prions are less efficient at spreading and causing disease within the host than wild-type prions. Collectively, these observations are consistent with the possibility that glycans and GPI anchors increase spreading efficiency and pathogenicity within the host, especially via peripheral sites of exposure.

### Structural comparisons to other infectious prion structures

Comparison of our current structure to the several other recently determined infectious prion structures indicates that it shares a number of general cross-sectional features with the rodent-adapted scrapie prion strains: an ordered core spanned by ~ 137 residues of a single PrP monomer; a staggered central interface between N- and C-lobes (Fig. 5); a disulfide arch with a low resolution tip; and outwardly projecting N-linked glycans and GPI anchor. However, beyond those similarities, the CWD fibril has many distinct features, including most broadly: the absence of a steric zipper at N-terminus; a buried lysine (K113) in the CLC; differences in the number and positions of β-strands; less helical twist with at least roughly twice the cross-over distance along the fibril axis; a ~ 180° rotation of the N-lobe relative to the C-lobe; and a more convoluted path of the PrP backbone within the N-lobe instead of more clearly defined N- and middle arches.

**Fig. 7:**
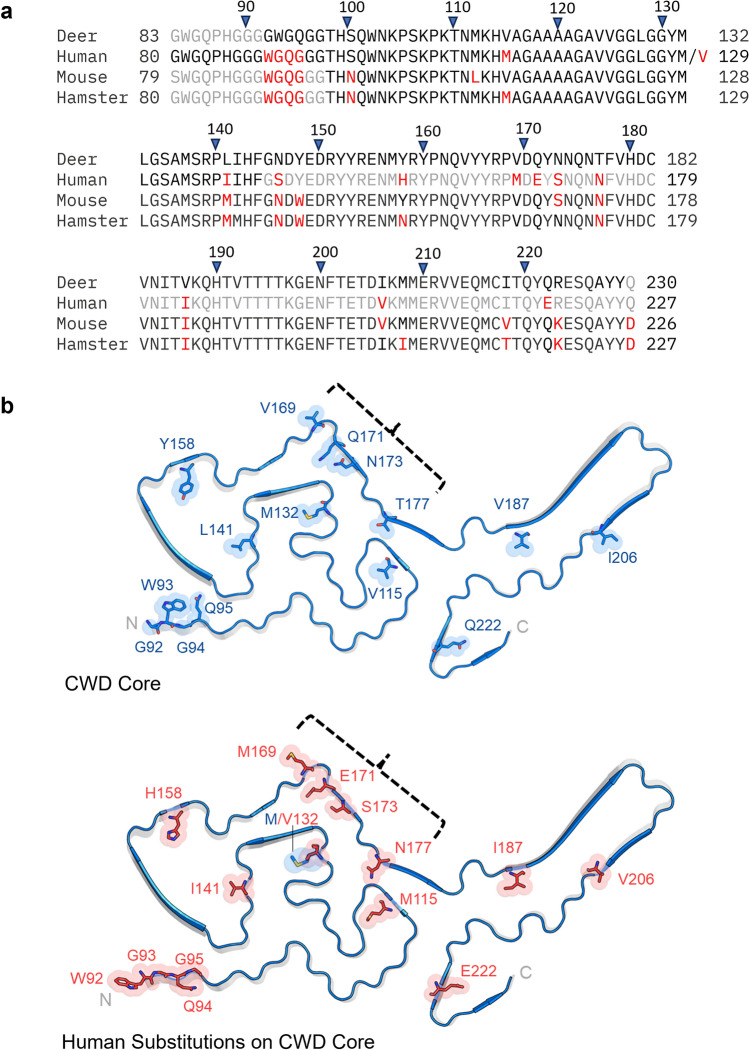
Species-associated sequence variations in regions overlapping core structures of ex vivo PrP^Sc^ fibrils. **a** Sequence alignment with deer numbering on top and individual species numbering on the sides. Positions at which the sequence differs from deer PrP are highlighted in red; residues outside the respective ordered core regions (based on structures of deer CWD (this paper), human GSS F198S [16], mouse a22L [20], and hamster 263K [25]) are shown in gray; (Clustal Omega program was used for this analysis [30]). **b** Positions at which human PrP residues (bottom, red) differ from the corresponding deer sequence (top, blue) superimposed on the CWD core cross-section; (mutation of the deer sequence was performed in Pymol/2.6.0). Brackets mark four sequence variations between deer and humans that have been shown collectively to strongly contribute to the barrier to transmission of CWD to transgenic mice that overexpress human PrP [27]. M/V132 marks the position that corresponds to position 129 in human PrP, where either M (like deer) or V is found normally

The center of the E motif (residues ~ 118–131) in the CWD fibril has packing of hydrophobic residues (Fig. 6a) as has been seen with the N-arches of most rodent-adapted strains [20, 21, 25, 31, 32]. The alternating alanine, valine, and glycine residues form a distinctive curvature at the heads of the N-arches of RML and 263K that is also seen in the E motif of CWD (Figs. 6a and S5a). Whereas the N-arch heads in the rodent-adapted strains form one half of the respective N- and C-lobe interfaces, the analogous hydrophobic patch in the CWD fibril does not directly mediate an interaction between the two lobes. The *trans* sidechain configuration of Y131 and M132 (deer numbering) observed in the CWD structure is similar to the analogous Y, M residue sidechains of RML [21, 32] and ME7 [31] but distinct from the *cis* configurations in 263K (outwardly facing with respect to the N-arch) [25] and a22L (inwardly facing) [20] (Figs. 6a and S5a). Striking conformational divergence occurs in residues flanking the shared ~ A118-G134 sequence of the CWD and rodent strains, resulting in formation of the CWD E motif instead of the extended stems of the N-arches of the rodent prions. The CWD hammer loop comprises residues ~ 138–163, while the analogous sequences form portions of the middle arch in the rodent PrP^Sc^ fibrils. However, unlike the middle arch, which is structurally conserved among the rodent fibrils (Fig. S5c), the hammer loop is unique. It presents only a small interface with the N-terminal stretch of residues, precluding the head of the loop from participating in a steric zipper as is seen with residues ~ 141–147 of the middle arch. In addition, the hammer loop is less oblong and does not exhibit a gradual taper, as residues 139 and 162 pinch the loop into a distinct neck; this allows for the hammer loop to be notably wider than the rodent middle arches. Despite increased flexibility, the disulfide arch of CWD most closely resembles the disulfide arches of ME7, wild-type RML, and aRML. The many conformational distinctions between the CWD and rodent prions could be influenced by differences in amino acid sequence within their respective PrP^Sc^ cores (Fig. 7), but the fact that murine prions strains with identical amino acid sequence have divergent conformations indicates that there can also be purely conformational determinants of prion strains [20, 31].

Differences between the CWD prion and the human GSS F198S fibril [16] are even more striking than those between CWD and the rodent-adapted prions. The GSS fibrils comprise 2–4 intertwined subfibrils, each with a much smaller and differently folded ordered core of residues 80–141 compared to 92–229 for CWD. F198S GSS has been transmitted to bank voles [40], which are unusually susceptible to a wide range of mammalian prions, but its transmissibility to humans, sub-human primates, or mice expressing human PrP^C^ is questionable [6]. In any case, all of the solved prion structures that are infectious enough to cause clinical disease on primary passage into susceptible hosts (other than bank voles) have ordered cores that either largely overlap both the N- and C-lobes of the CWD and rodent prion structures, or partially overlap the N-lobe. Several high-resolution structures are available for synthetic PrP amyloid fibrils with ordered cores that overlap the C-lobe (disulfide arch region) of the CWD and rodent prion structures [53, 54], but such structures are not known to be infectious. Although solution of many more prion structures will be needed to understand the full range of transmissible PrP prions, this new CWD structure provides the first naturally occurring example of highly infectious prion with an overall N-lobe + C-lobe PIRIBS architecture.

### CWD strains and cervid PrP sequence variation

Although our sample size was limited to one WTD, the case material should be broadly representative of chronic wasting disease in North American cervid species. This deer was, as noted above, expressing the “wild-type” PrP amino acid sequence that is most common among WTD [17, 47] and also is the most susceptible of the cervid PrP variants. Thus, this isolate may represent a widespread strain of prion affecting cervids in North America, if not the most common strain. Moreover, the sequence of our subject deer is identical to the most common sequence in mule deer (*Odocoileus hemionus*), North American moose (*Alces alces*), caribou (*Rangifer tarandus*), and other nonnative species in the Subfamily Capreolinae [17]. This sequence is only one amino acid (E226) different from the most common sequence in wapiti (*Cervus canadensis*, or North American elk) and the same or close to the sequence of other species in the Subfamily Cervinae [17]. Consequently, the inferences that can be made from our findings may extend well beyond the single deer represented in our current study.

### Potential mechanisms of protective WTD PrP variants

The PrP molecule comprising this CWD prion fibril structure was of the most common “wild-type” sequence. However, multiple other WTD PrP sequences have been identified (summarized in [2, 45]). Notably, epidemiological studies suggest that the Q95H and G96S substitutions are partially protective against CWD [35, 43]. In our current CWD structure, these two residues are near the N-terminus where the Q sidechains on adjacent chains in the PIRIBS stack likely form a stabilizing H-bonded amide zipper along the axis of the fibril. However, such amide zippers would not be possible between stacked histidine sidechains. The possible impacts of the G96S substitution remain unclear to us with respect to accommodation within the CWD core structure. However, variants may also affect conversion efficiency by altering the behavior of PrP^C^ or a conversion intermediate independent of explicit effects on the stability of a hybrid PrP^Sc^ product.

### Potential mechanisms for the WTD CWD to human species barrier

As noted above, considerable evidence indicates that CWD is difficult to transmit to humans or human surrogates. For deer CWD to be infectious for humans, human PrP molecules must be incorporated onto CWD prions in a way that enables further propagation. Moreover, this process must occur with sufficient efficiency to outpace any clearance mechanisms and allow PrP^Sc^ accumulation to clinically relevant levels within the natural lifespan of the host. Analogously to what we have shown previously in the case of the hamster 263K-to-mouse transmission barrier [25], knowledge of the high-resolution CWD structure now allows us to consider why amino acid residue differences between deer and humans might represent obstructions to CWD-induced conversion of human PrP. Human PrP differs at 15–16 positions (depending on the human M/V polymorphism at residue 129) within the sequence comprising the ordered CWD fibril core (Fig. 7). Although each of these sequence differences should ultimately be scrutinized, here we will restrict our discussion to differences that have been shown empirically to diminish CWD-induced conversion of human PrP^C^. For example, a cluster of four sequence differences is found within a segment corresponding to deer residues 169VDQYNNQNT177, the so-called the β2-α2 ‘rigid’ loop within PrP^C^ (reviewed in [28]). Although transgenic mice expressing wild-type human PrP^C^ have been found to be resistant to CWD, mice expressing a chimeric PrP^C^ in which this cervid sequence was substituted into the human PrP^C^ background were highly susceptible [27]. Inspection of the CWD structure reveals that three of these four substitutions are of residues with inwardly oriented sidechains that might not fit well into the spaces dictated by the CWD template (Fig. 7b, brackets). One of these, a glutamate (E) for glutamine (Q) substitution (at deer residue 171), would require that a charged sidechain be buried in a cavity without a compensating cationic group. Another previous protein misfolding cyclic amplification (PMCA) study provided evidence that homology between the deer M132 (Fig. 7b) and the corresponding human residue at position 129 (M or V) improved the ability of WTD CWD to incorporate human PrP [5]. Interestingly, the M132 sidechain projects toward the inside of a loop of the CWD E motif, a site in which accommodation of a wider branched valine sidechain might be difficult. Further study will be needed to fully elucidate the mechanisms of these and other potential barriers to CWD transmissions into humans.

### Weaknesses of this study

Due in part to the often-clumped nature of CWD fibril preparations, our single-particle acquisition process involved manual picking of suitably isolated regions of single fibrils for imaging. Thus, the possibility remains that this process excluded particles that may be biologically relevant but too clumped or variable to be resolved in the final reconstruction of the 3D electron density map. Also, this structure represents prions isolated from a single deer, making it important to remain aware of the possibility of deer-to-deer variation in CWD structure.

## Conclusion

The WTD CWD prion fibril structure reported here is the first associated with a natural prion disease in a mammalian host expressing wild-type PrP molecules. Although the CWD fibril has a cross-β amyloid core architecture, as do those of experimental rodent-adapted scrapie prion strains and the human GSS F198S amyloid, striking differences from all previously determined PrP fibril structures were found. Presumably, these distinctive CWD structural features provide molecular foundations for the biological properties of this highly, and naturally, transmissible mammalian proteinopathy. Our expanding knowledge of prion structures should aid in the rational development of approaches to prion disease prevention, diagnostics, and therapeutics, and may help explain past shortcomings based on rodent models that in retrospect may not have faithfully represented some of the natural processes of interest. It is notable that previous attempts to develop vaccines against CWD in cervids failed to be protective, and at least in one case, had the opposite effect [41, 57]. In light of our new CWD structure, we speculate that a possible explanation for adverse effects of a given vaccine could be that antibody binding to the sides, rather than the terminal seeding surfaces, of prion fibrils might promote fragmentation, but not the total destruction, of prions. This would increase prion particle number without inhibiting prion propagation. Thus, with vaccines, as well as small-molecule inhibitors, it may be most important to target the binding and conversion of PrP molecules at the growing tips of prion assemblies.

## Supplementary Information

Below is the link to the electronic supplementary material.

Supplementary file1 (DOCX 1805 KB): Figs. S1–S4 and Table S1

## Data Availability

The cryo-EM density map of the CWD PrP^Sc^ fibrils (EMD ID 47020 or 47021) is available from the Electron Microscopy Data Bank. Atomic models of the fibrils^Sc^ without (PDB ID 9DMY) and with (9DMZ) the first N-linked N-acetylglucosamine residues at N184 and N200 are available at the Protein Data Bank.
