# Supplementary material for: Cryo-EM structure of a natural prion: chronic wasting disease fibrils from deer
Source: Acta Neuropathol. 2024 Oct 24;148(1):56. doi: 10.1007/s00401-024-02813-y (PMC11502585; doi:10.1007/s00401-024-02813-y)
Supplement: Supplementary file 1 — Supplementary file1 (DOCX 1805 KB): Figs. S1–S4 and Table S1 [file 401_2024_2813_MOESM1_ESM.docx]

**SUPPORTING INFORMATION**


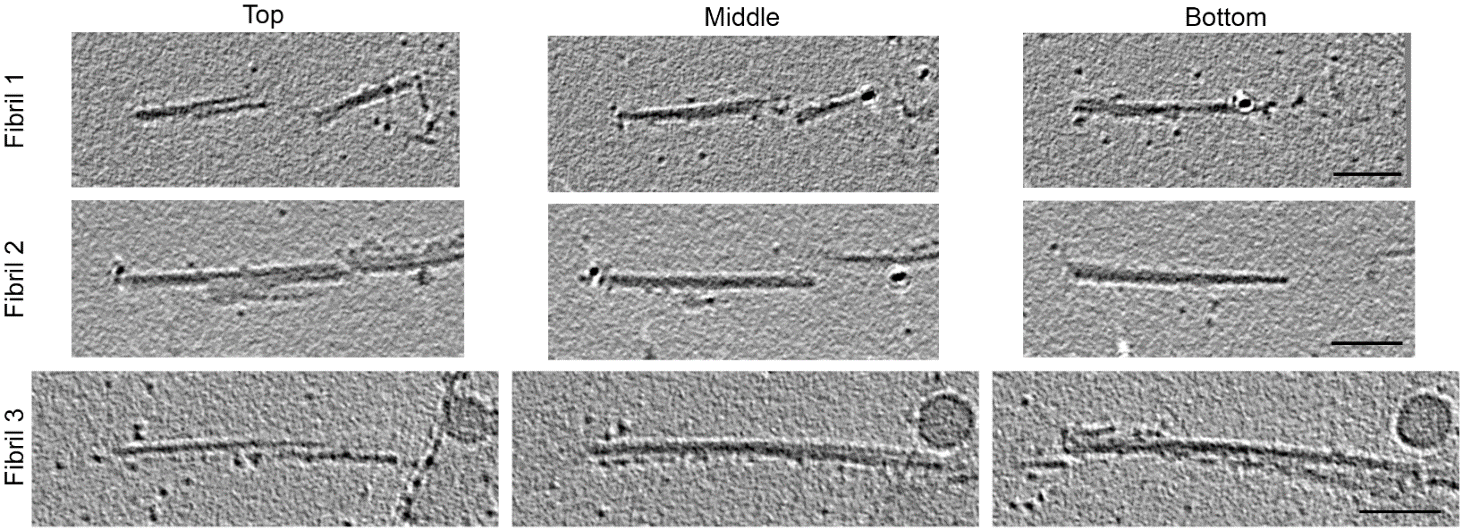


**Fig. S1** Cryo-EM tomography of CWD fibrils. Representative images of three fibrils showing top, middle and bottom slices through the tomograms, indicating left-handed twist in each fibril. Scale bars: 50 nm.


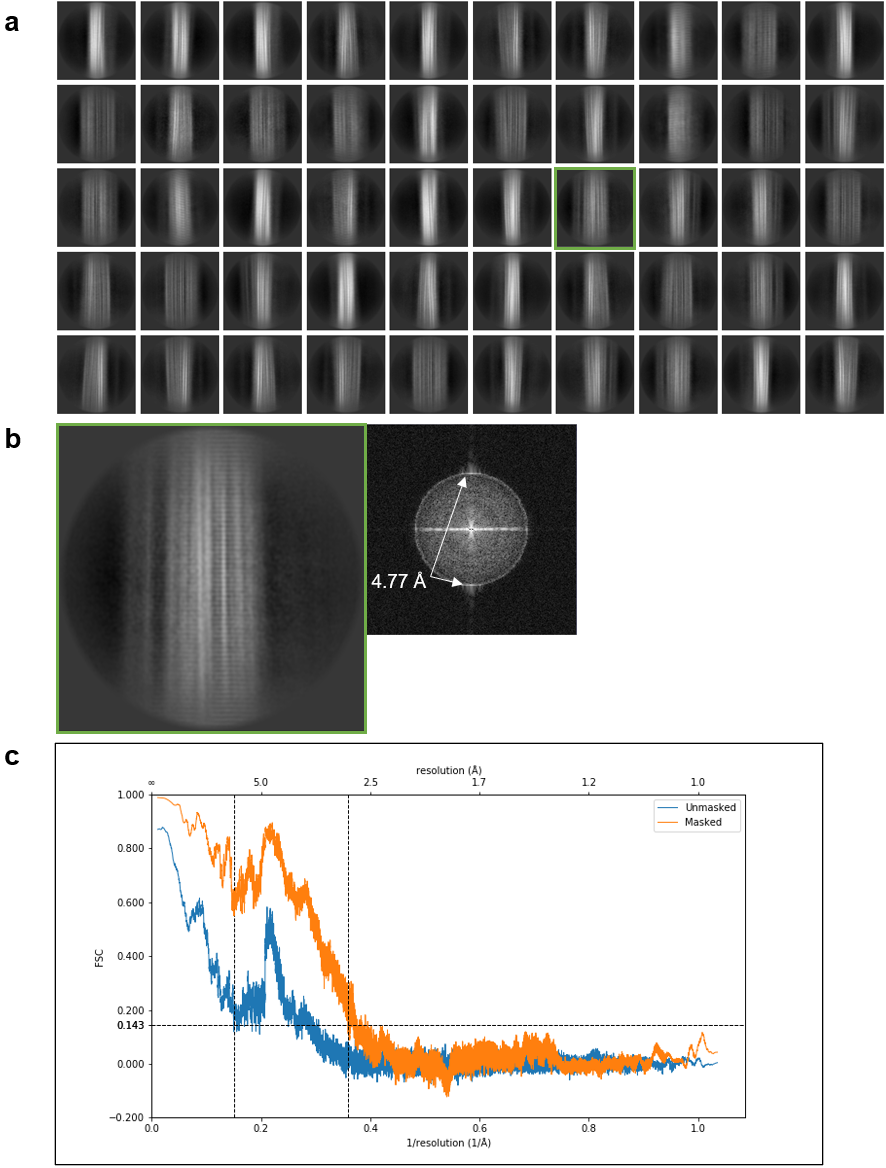


**Fig. S2 a** Representative 2D class averages showing lateral views of CWD fibril segments. **b** An enlargement of the class average boxed in green in **a** allows easier visualization of the fine horizontal bands running perpendicular to the fibril axis. Right panel: fast Fourier transform of 2D class average boxed in green indicating signals at ~4.77 Å that correspond to the spacing between the bands. **c** Fourier shell correlation plots of masked and unmasked models indicating, from the masked trace, a maximal resolution of 2.8 Å.


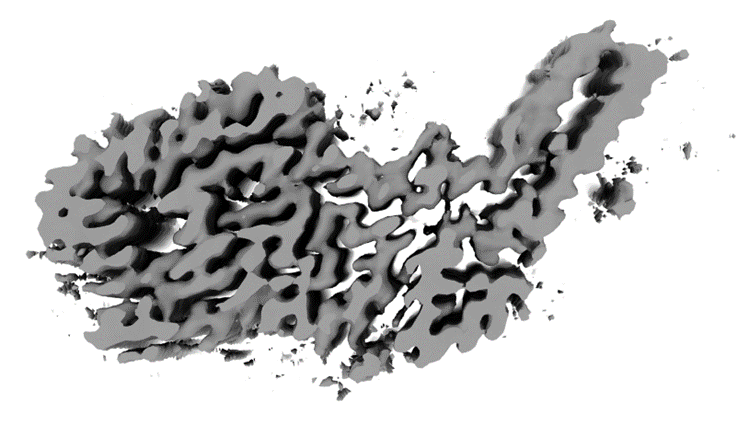


**Fig. S3** Cross-section of the unsharpened 3D map from auto-refinement in Relion. Peripheral densities can be clearly identified in association with the prion core.


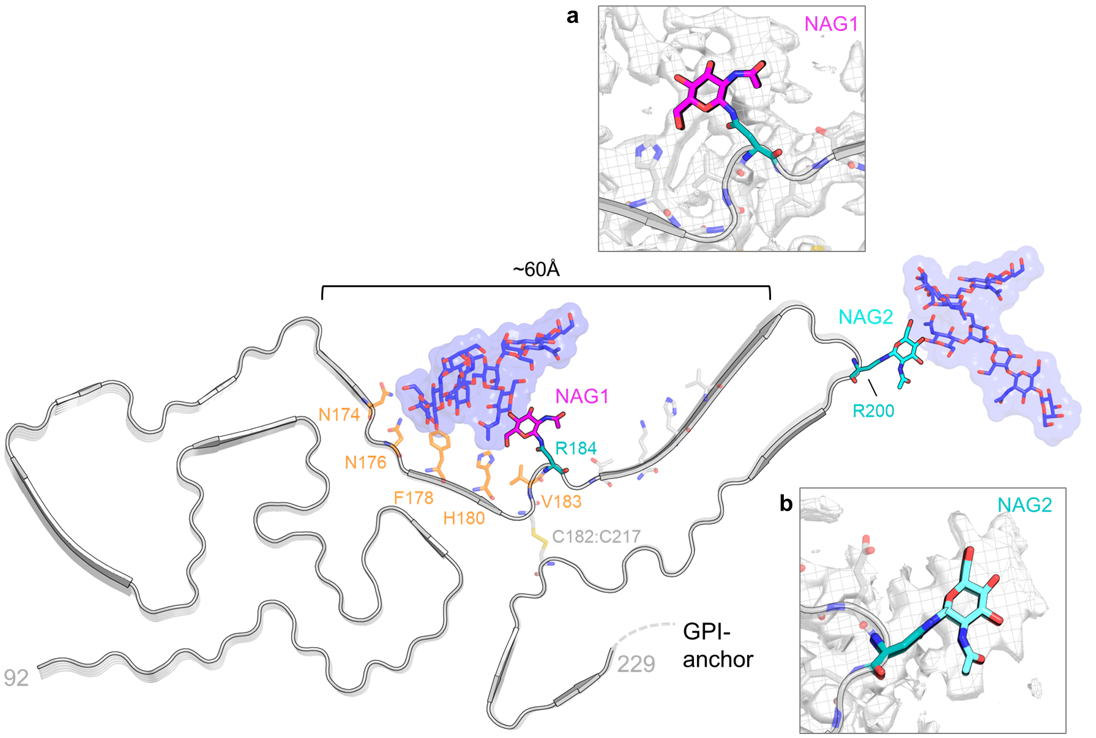


**Fig. S4** Glycosylation of CWD PrP^Sc^. Overview of the CWD structure with the two resolved N-acetylglucosamine residues NAG1 (magenta) and NAG2 (cyan). Representative tri-antennary glycans were modeled onto the NAG1 and NAG2 residues with CHARMM-GUI [22] and are depicted in dark blue (carbons) with red (oxygens). Residues forming potential interactions with the N-linked glycan attached to R184 are colored in orange. Width of the cleft between the N- and C-lobes is ~60 Å. Inset (**a)**: structure of NAG1 (magenta) on R184 (teal) against a mesh/surface plot of the cryo-EM density (grey). Inset (**b**): structure of NAG2 (cyan) on R200 (teal) against cryo-EM density.


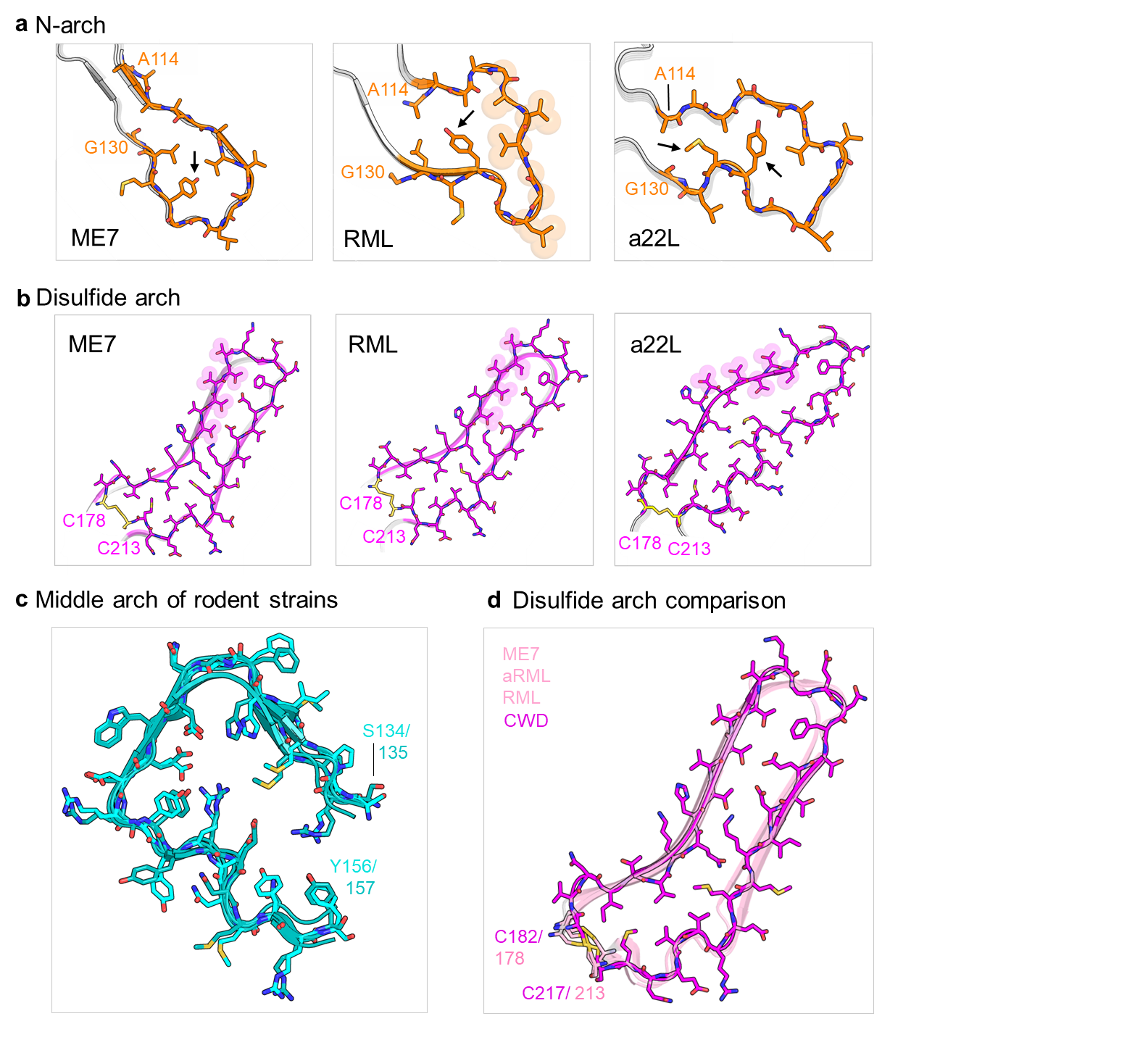


**Fig. S5** Rodent-adapted prion strain comparison of major PrP^Sc^ structural motifs. **a** Residues 114-130, which comprise the N-arch in mouse strains ME7, RML and a22L. Highlighted residues span 119-124 and arrow marks Y120. **b** Disulfide arches of three mouse strains (ME7, RML and a22L), which begin at the disulfide bond formed by residues C178 and C213. The prominent stretches of 5 closely spaced threonine residues are highlighted. **c** Overlay of middle arches from five rodent strains (ME7, aRML, RML, a22L and 263K). Hamster residues are colored in teal and mouse residues are colored in cyan. **d** Comparison of CWD disulfide arch (magenta) with the disulfide arches of three mouse strains (pink; ME7, aRML and RML)

**Table S1** Excel file with a summary of cryo-EM tomography twist results from all fibrils analyzed.

| **Tomograph** |  | **Twist** |
| --- | --- | --- |
| CWD81K | 1 | L |
|  | 2 | L |
|  | 3 | L |
| CWD81K_30 | 1 | L |
| CWD81K_31 | 1 | L |
|  | 2 | L |
| CWD81K_37 | 1 | L |
|  | 2 | L |
|  | 3 | L |
| CWD81K_39 | 1 | L |
| CWD81K_41 | 1 | L |
|  | 2 | L |
| CWD81K_42 | 1 | L |
|  | 2 | L |
| CWD81K_15 | 1 | L |
|  | 2 | L |
| CWD81K_19 | 1 | L |
| CWD81K_1 | 1 | L |
| CWD81K_24 | 1 | L |
|  | 2 | L |
| CWD81K_27 | 1 | L |
| CWD81K_40 | 1 | L |
| CWD81K_4 | 1 | L |
|  | 2 | L |
